# P-1381. Predictive Factors Of Non-Favorable Outcomes In Extrapulmonary Tuberculosis

**DOI:** 10.1093/ofid/ofaf695.1568

**Published:** 2026-01-11

**Authors:** Fatma Hammami, Khaoula Rekik, Amal Chakroun, Makram Koubaa, Fatma Smaoui, Chakib Marrakchi, Mounir Ben Jemaa

**Affiliations:** Infectious Diseases Department, Hedi Chaker University Hospital, University of Sfax, Tunisia, Sfax, Sfax, Tunisia; Infectious Diseases Department, Hedi Chaker University Hospital, University of Sfax, Tunisia, Sfax, Sfax, Tunisia; Infectious Diseases Department, Hedi Chaker University Hospital, University of Sfax, Tunisia, Sfax, Sfax, Tunisia; Infectious Diseases Department, Hedi Chaker University Hospital, University of Sfax, Tunisia, Sfax, Sfax, Tunisia; Infectious Diseases Department, Hedi Chaker University Hospital, University of Sfax, Tunisia, Sfax, Sfax, Tunisia; Infectious Diseases Department, Hedi Chaker University Hospital, University of Sfax, Tunisia, Sfax, Sfax, Tunisia; Infectious Diseases Department, Hedi Chaker University Hospital, University of Sfax, Tunisia, Sfax, Sfax, Tunisia

## Abstract

**Background:**

Extrapulmonary tuberculosis (EPT), a significant share of tuberculosis cases, is often associated with diagnostic delays and variable clinical outcomes. Identifying factors that predict non-favorable outcomes is essential to improve patient care and prognosis. We aimed to study the predictive factors of non-favorable outcomes in EPT cases.

**Methods:**

We conducted a retrospective study including all patients hospitalized in the infectious disease department for EPT between 1996 and 2024. Non-favorable outcome was defined by the occurence of death, relapse or sequelae.

**Results:**

We encountered 618 cases, among whom 106 had non-favorable outcomes (17.2%). Fever (67% vs 51.8%; p=0.004), asthenia (70.8% vs 51.2% ; p< 0.001), anorexia (62.3% vs 40.7%; p< 0.001) and weight loss (50% vs 38.5%; p=0.028) were significantly more frequent among non-favorable outcome cases. Neuromeningeal tuberculosis (25.5% vs 11.2% ; p< 0.001), osteoarticular tuberculosis (20.8% vs 12.7% ; p=0.031) and miliary tuberculosis (19.8% vs7.4% ; p< 0.001) were significantly associated with non-favorable outcomes. Elevated C-reactive protein levels were noted more frequently among cases with non-favorable outcomes (25[5-70] mg/l vs 8[4-45] mg/l; p=0.012). Previous medical history of cancer (7.5% vs 4.7% ; p=0.22), diabetes mellitus (4.8% vs 5.7% ; p=0.71) and *Human immunodeficiency virus* infection (1.9% vs 0.6% ;p=0.20) were noted among cases with non-favorable and favorable outcomes. Seperate tablets of antitubercular therapy (70.8% vs 57.9% ;p=0.015) and corticosteroid therpy (37.1% vs 15.7% ; p< 0.001) were more frequently prescribed for cases with non-favorable outcomes. No significant difference was noted concerning the age or the gender.

**Conclusion:**

The presence of cardinal symptoms of tuberculosis, eleaved C-reactive protein levels, seperate tablets of antitubercular therapy and corticosteroid therpy, mainly among severe forms of tuberculosis including neuromeningeal, osteoarticular and miliary tuberculosis, were predictor factors of non-favorable outcomes among cases of EPT. Prompt diagnosis of severe forms and optimization of treatment regimens are critical to reducing poor outcomes in EPTB.

**Disclosures:**

All Authors: No reported disclosures

